# Network Meta-Analysis Using R: A Review of Currently Available Automated Packages

**DOI:** 10.1371/journal.pone.0115065

**Published:** 2014-12-26

**Authors:** Binod Neupane, Danielle Richer, Ashley Joel Bonner, Taddele Kibret, Joseph Beyene

**Affiliations:** 1 Department of Clinical Epidemiology and Biostatistics, McMaster University, MDCL 3200, 1280 Main Street West, Hamilton, Ontario, Canada, L8S 4K1; 2 Department of Mathematics and Statistics, McMaster University, HH 218, 1280 Main Street West, Hamilton, Ontario, Canada, L8S 4K1; University of North Carolina at Charlotte, United States of America

## Abstract

Network meta-analysis (NMA) – a statistical technique that allows comparison of multiple treatments in the same meta-analysis simultaneously – has become increasingly popular in the medical literature in recent years. The statistical methodology underpinning this technique and software tools for implementing the methods are evolving. Both commercial and freely available statistical software packages have been developed to facilitate the statistical computations using NMA with varying degrees of functionality and ease of use. This paper aims to introduce the reader to three R packages, namely, *gemtc*, *pcnetmeta*, and *netmeta*, which are freely available software tools implemented in R. Each automates the process of performing NMA so that users can perform the analysis with minimal computational effort. We present, compare and contrast the availability and functionality of different important features of NMA in these three packages so that clinical investigators and researchers can determine which R packages to implement depending on their analysis needs. Four summary tables detailing (i) data input and network plotting, (ii) modeling options, (iii) assumption checking and diagnostic testing, and (iv) inference and reporting tools, are provided, along with an analysis of a previously published dataset to illustrate the outputs available from each package. We demonstrate that each of the three packages provides a useful set of tools, and combined provide users with nearly all functionality that might be desired when conducting a NMA.

## Introduction

Network meta-analysis (NMA), also known as multiple treatment comparison (MTC) or multiple treatment meta-analysis (MTM), has been increasingly used in recent years [1]–[3] to simultaneously compare the effects of multiple treatments on a health outcome. NMA is being rapidly adopted across a wide range of health research areas [4]. Researchers looking to undertake a NMA in their field will find familiarity in the systematic processes of searching, selecting and grading contributing studies, as is required for a standard meta-analysis [1]. However, the additional analysis complexities involved with a NMA requires the user to be aware of model considerations, diagnostic tools, and reporting styles.

NMA can be performed either under a frequentist or a Bayesian framework, and several models have been proposed under both frameworks [5]–[9]. Network meta-analysts must select a modeling approach and are advised to explore the differences between the frequentist and Bayesian approaches [10]. The Bayesian approach is more frequently used [1], [3] as it can produce estimates of rank probabilities (the probability that each treatment to be the best, second best, and so on). After making several model-based choices, diagnostic processes must be undertaken to verify if the model was appropriate. These approaches must assess heterogeneity and inconsistency, two assumptions underlying any NMA that are highly influential to the results. Methods of identifying and dealing with these issues are explored extensively in the NMA literature [11]–[16]. It is important to publish NMA results clearly and completely. Methods for reporting NMA results are discussed at length in Bafeta et al [1]. Displaying the network, presenting relative effects and rank probabilities are an important part of reporting NMA results.

There are several statistical programs available that can implement the various steps required to carry out a NMA. Frequentist models can be implemented using commercial programs such as SAS and STATA. Freely available Bayesian software programs such as OpenBUGS, WinBUGS, or JAGS can be used to conduct Bayesian NMA, but they require developing a program code (or modifying pre-existing codes) that can be quite involved. In addition, some of the plotting tools of interest to NMA researchers are not incorporated into these programs. The statistical software program R is freely available and popular among statisticians because it is open source, allowing for the implementation of new statistical methods almost instantaneously through the creation of packages. R interfaces with all three Bayesian software programs mentioned above to conduct network meta-analyses with the use of appropriate packages. The user is not required to program in OpenBUGS, WinBUGS or JAGS in order to implement these packages, minimizing the programming required of the user. By combining the functionality of a few packages, almost all desired outputs can be obtained in R.

Recently, three packages, *gemtc* (http://cran.r-project.org/web/packages/gemtc/index.html), *pcnetmeta* (http://cran.r-project.org/web/packages/pcnetmeta/index.html), and *netmeta* (http://cran.r-project.org/web/packages/netmeta/index.html), have been developed specifically for network meta-analysis in the *R* environment, allowing users to perform NMA with minimal programming. At the time of writing (July 2014), these are the only packages developed specifically for performing NMA that we identified. Each can automatically generate and run the analysis model with minimal programming required by users. The first two packages perform the analysis under the Bayesian framework and the third performs under the frequentist framework.

The purpose of this paper is to present a comparative review of three R packages, namely, *gemtc, pcnetmeta*, and *netmeta* with respect to functionality, flexibility and ease of use. This guide is designed to inform new users of NMA who are familiar with the R environment and would like to find out which packages might suit their needs. If researchers know the statistical choices they want to make, this paper will help them to determine how to do it in R.

This paper is organized as follows. The Methods section below describes the approach we followed to identify and explore the three packages. The Results section summarizes our findings, including an analysis of real data using each package. The last section provides conclusions about our investigation.

## Methods

### R packages for NMA

We searched the Comprehensive R Archive Network (CRAN) for any contributed R packages written primarily for NMA. Three R packages met this requirement: *gemtc*, *pcnetmeta*, and *netmeta*. Although we found other packages with some applications for NMA, including *metaphor* and *mvmeta*, we did not consider these packages as they are written for general purpose meta-analysis (univariate and multivariate, respectively). The *gemtc* package synthesizes evidence on the relative effects of multiple treatments by fitting generalized linear model (GLM) under a Bayesian framework. The *pcnetmeta* synthesizes probabilities of events in all treatments from a network of trials using a multivariate meta-analysis technique, also under a Bayesian framework. The *netmeta* package is based on graph theory methodology to model the relative treatment effects of multiple treatments under a frequentist framework.

In the next section, we present a more detailed general introduction to network meta-analysis, especially concerning the objective of NMA, the input data, and the methodology. Details about the specific data input and analysis options, statistical models, methods, and formulations used in the three packages can be found in the respective reference manuals and original articles: van Valkenhoef et al [17] for *gemtc*, Zhang et al [18] for *pcnetmeta*, and Konig et al [19] and Krahn et al [20] for *netmeta*.

### Methodological and statistical aspects of NMA

NMA enables investigators to compare the effects of multiple health care interventions including treatments that were not previously compared in head-to-head trials. Additionally, combining indirect and direct evidence can sometimes provide more precise estimates of treatment effects to support decision-making.

Depending on the type of outcome (e.g., binary, count, continuous), the input aggregate data set can be either *arm-level* (e.g., observed number of events and number of patients randomized in a treatment arm in each trial for a binary outcome such as incidence of diabetes) or *contrast-level* (e.g., estimate of the relative treatment effect such as log-odds ratio and its standard error for the binary outcome for any two treatments in a trial). There are two broad statistical inference frameworks that are typically used in NMA: a frequentist versus a Bayesian approach. The Bayesian framework is quite flexible and allows incorporating prior information on model parameters and captures uncertainties comprehensively. In addition, one could make direct probabilistic statements about parameters of interest.

For the results of NMA to be valid, the network is assumed to maintain transitivity (potential modifiers of treatment effects are similarly distributed across trials) and consistency (indirect effect estimates are consistent with that of direct effects), while interpretation of the treatment effects is more straightforward if they are also homogeneous across trials [21]. Therefore, a careful evaluation of clinical and methodological heterogeneity across trials is important to make sure that the network maintains transitivity (i.e., includes trials with similar patients and trial characteristics within and across trials). The presence of heterogeneity and inconsistency in the network can be quantified and assessed statistically, for which different methods have been proposed [11]–[16]. If there is unexplained heterogeneity – identified through clinical or statistical investigations - a random-effects rather than a fixed-effect model is preferred. It is a common practice in the NMA literature to assume a common heterogeneity for all treatments effects under random-effect assumption. Methods have been proposed to account for inconsistency if suspected [14]. Assessment of goodness-of-fit may also help to identify more appropriate model (e.g., fixed vs. random-effects) for the data [6]. Akaike information criteria (AIC) and Deviance information criteria (DIC) are widely used criteria to assess goodness-of-fit of the models in frequentist and Bayesian frameworks, respectively. Detailed reviews about assessing and dealing with heterogeneity [22], [23] and inconsistency [24] in a network and choice of frequentist or Bayesian frameworks for NMA [25] are provided in great details in the first book on network meta-analysis [26].

Our review of the three R packages reflects the methodological and statistical aspects of NMA described above. To summarize, an analyst begins with an exploration of the network data, and proceeds with proposing and assessing a model for the data. The model assumptions and fit are assessed with diagnostic procedures to come up with a “final” model, which will then be used to generate and interpret results.

With this process in mind, we reviewed the available features or capabilities of these packages with respect to conducting a NMA: importing and preparing data, creating a model, detecting and dealing with heterogeneity and inconsistency and assessing model fits, and obtaining estimates of relative effects or rank probabilities. The authors of the respective packages were consulted to verify the accuracy of Tables 1–4. We also used each package to perform network meta-analysis of publicly available data on the incidence of diabetes [27]. In particular, this data set was selected for illustration because it represents a typical network consisting of comparison of the effects of 6 treatments in 22 trials, close to the median numbers of 6 treatments and 21 trials, respectively, on a binary outcome, the most common outcome type, in the NMA literature [3]. The network includes multi-arm studies and there is an evidence of inconsistency in the network, thus providing an opportunity to see how each of the packages identifies and deals with this common issue. The output from each package is included to provide visuals of the reporting tools available.

**Table 1:** Data input and network plotting functionality from NMA R packages *gemtc, pcnetmeta* and *netmeta.*

Tasks	Features	gemtc	pcnetmeta	netmeta
**Forms of input data**	Arm-level data	✓	✓	✗
	Contrast-level data	✓	✗	✓
	Accepts multi-arm (≥3) trials	✓	✓	✓
**Types of outcome data that can be analyzed**	Binary	✓	✓	✓
	Count	✓	✗	✓
	Continuous	✓	✗	✓
	Survival	✓	✗	✓
**Extracts descriptive measures**	Total number of studies	✓	✗	✓
	Total number of multi-arm studies	✓	✗	✓
	Total number of participants	✓	✗	✗
	Total number of treatments	✓	✗	✓
**Network plot and options**	Network plot	✓	✓	✓
	Add node labels	✓	✓	✓
	Node size reflects network characteristic	✗	✓ User can specify, default by # studies using the treatment	✗
	Edge thickness reflects network characteristic	✗	✓ Number of studies making this comparison	✓ Inverse standard error of aggregated direct treatment effects

**Table 2:** Modeling options from NMA R packages *gemtc, pcnetmeta* and *netmeta.*

Tasks	Features	gemtc	pcnetmeta	netmeta
**NMA Model**	Based on	Generalized Linear Models [6], [17]	Multivariate methods [18]	Graph theory [32]
**Model options regarding the Homogeneity assumption**	Fixed-effect model	✓	✓	✓
	Random-effect model with a common heterogeneity parameter	✓	✓	✓
	Random-effect model with different heterogeneity parameters	✗	✓	✗
**Model options regarding the Consistency assumption**	Consistency model	✓	✓	✓
	Inconsistency model	✓	✗	✓
**Inclusion of covariates**	Meta-regression	✗	✗	✗
**Estimation framework**	Frequentist	✗	✗	✓
	Bayesian	✓	✓	✗
***Bayesian Modeling***				NA
*Prior distributions for baseline and relative effect parameters*	Default distribution and parameter values	✓ Normal distribution, heuristic initial values	✓ Normal distribution, heuristic initial values	
	Option for user-specified distribution and parameter values	✓ Restricted to specifying variance	✓ Restricted to Normal distribution	
*Prior distribution for heterogeneity parameters*	Default distribution and parameter values	✓ Uniform distribution, heuristic initial values	✓ Inverse-Gamma distribution, specific values	
	Option for user-specified distribution and parameter values	✓ Uniform or Gamma distribution, specify values	✓ Inverse-Gamma or Wishart distribution, specify values	
*Markov Chain Monte Carlo (MCMC) Sampler*	WinBUGS	✓	✗	
	OpenBUGS	✓	✗	
	JAGS	✓	✓	
*Control over posterior samples*	Total iterations	✓	✓	
	Adaptation phase	✓	✓	
	Burn-in phase	✓	✓	
	Thinning	✓	✓	
*Model convergence diagnostics*	Option for multiple chains	✓	✓	
	Time-series plot	✓	✓	
	Trace plot	✓	✗	
	Brooks-Gelman-Rubin (BGR) diagnostic test	✓	✗	

**Table 3:** Assumption checking and diagnostic testing functionality from NMA R packages *gemtc, pcnetmeta* and *netmeta*.

Tasks	Features	gemtc	pcnetmeta	Netmeta
**Assessing Heterogeneity**	Visual inspection - forest plot	✓	✗	✓
	Pairwise statistics	✓	✗	✓
	Global statistics	✓	✗	✓
**Assessing Inconsistency**	Visual inspection - forest plot of direct vs. indirect	✓	✗	✗
	Visual inspection – heat map	✗	✗	✓ (net heat plot)
	Consistency statistics	✓	✗	✓
	Back-calculation	✓	✗	✗
	Node-split/decomposition	✓	✗	✓
**Goodness of model fit**	Deviance information criterion (DIC)	✓	✓	NA
	Akaike information criterion (AIC)	NA	NA	✗

**Table 4:** Inference and reporting tools available from NMA R packages *gemtc, pcnetmeta* and *netmeta.*

Tasks	Features	gemtc	pcnetmeta	netmeta
**Available summary effect measures for output**	Relative risk (RR)	✗	✓	✓
	Odds ratio (OR)	✓	✓	✓
	Risk difference (RD)	✗	✓	✓
	Absolute risk (AR)	✗	✓	✗
	Mean difference (MD)	✓	✗	✓
	Standard mean difference (SMD)	✗	✗	✓
	Arcsine difference (AS)	✗	✗	✓
	Event rates	✗	✓	✗
**Estimated effect measures**	Listed with confidence/credible intervals	✓	✓	✓
	Available in a table format	✗	✓	✓
	Available in a forest plot with specified reference treatment	✓	✗	✓
	Plot of estimated event rates with credible intervals	✗	✓	✗
	Density plot of posterior samples	✓	✓	NA
**Rank probabilities**	Estimates of ranks probabilities	✓	✓ (1^st^ only)	NA
	Rank probabilities plot (rankogram)	✓	✗	NA
	SUCRA	✗	✗	NA

Abbreviations and notations: NA not applicable; SUCRA sum under the cumulative ranking probabilities.

## Results

Tables 1 to 4 summarize the important features of NMA that are available in one or more of the latest versions of the *gemtc* (version 0.6, released on 2014-03-11) [28], *pcnetmeta* (version 1.1, released on 2014-03-09) [29], and *netmeta* (version 0.5-0, released 2014-06-24) [30] packages.

### Introduction to the packages

#### *gemtc* (version 0.6, released on 2014-03-11)

The package *gemtc* provides a comprehensive set of tools to perform NMA in a Bayesian setting. Arm- or contrast-level network data can be input of the four common outcome types (binary, continuous, count or survival). It models the relative effects (e.g., log-odds ratio) fitting a generalized linear model (GLM) under the Bayesian framework by linking to JAGS, OpenBUGS or WinBUGS as first described by Lu and Ades [7], and extended by others [6], [12], [28], [31]. Important features of this package include its ability to model heterogeneity and inconsistency [6], [12], [17]. It provides flexibility in modeling as users can specify different likelihood and link functions, priors for hyper parameters, and several Markov-Chain Monte-Carlo (MCMC) sampling options. Estimates of relative treatment effects can be plotted via forest plots and that of rank probabilities can be plotted via rankograms.

#### *pcnetmeta* (version 1.1, released on 2014-03-09)

The package *pcnetmeta* provides a small set of easy-to-use tools to conduct a Bayesian NMA for the simple case of binary data where inconsistency is disregarded. The package reads in arm-based summary data of binary outcomes and models the event rates (i.e. probabilities of success) in different treatments using multivariate Bayesian hierarchical mixed model approach [18]. The package interfaces with JAGS software to conduct MCMC sampling. Estimates of relative treatment effects such as relative risks (RR), risk difference (RD) or odds ratio (OR) can be calculated for any two treatments. This package can be used for the detection as well as incorporation of (common or differential) heterogeneity of event rates across trials; however, it does not provide any function for identifying or incorporating inconsistency in the analysis. Outputs include a confidence interval plot of the estimated event rates and posterior density plots.

#### *netmeta* (version 0.5-0, released 2014-06-24)

The package *netmeta* provides a comprehensive set of functions for conducting a NMA in a frequentist setting. The package employs graph theory methodology presented in [32]. Contrast-level summary data (e.g. log-odds ratio) are input, so all types of outcome data can be meta-analyzed in this package. The modeling process provides flexible options for the incorporation of heterogeneity and inconsistency in the estimation. A unique feature of this package is the *netheat* function, which employs a heatmap plot [20] for the detection of inconsistency.

### Summary of Features

Table 1 presents features regarding the compatibility of each package with different data types and data management steps, as well as their ability to describe and explore the network data. This table is fundamental to determine which of the packages are compatible with a researcher's data. NMA may use either arm-level or contrast-level summary measures collected from various trials. The NMA packages are therefore designed to employ both or at least one of the two data formats. The *gemtc* package is currently the only package that can accommodate both input types. Binary outcomes, the most common in NMA literature [3], are handled by all three packages, and it is the only outcome type that *pcnetmeta* can handle. Investigation of the network geometry is an important exploratory step; each package includes network plotting functions whereby some network characteristics can be displayed visually.

Table 2 shows the availability of modeling features for the packages. Each of the three NMA packages uses a different modeling approach. The technical details of these approaches are available in the referenced papers. Modeling options that relax the assumptions of homogeneity and consistency are important considerations. In reality, heterogeneity and inconsistency may be present in networks of evidence and decisions about modeling in such circumstances are key considerations for NMA researchers. While both Bayesian and frequentist NMAs are being published increasingly, the Bayesian framework is being used more often [3]. Characteristics of the Bayesian modeling process are summarized in Table 2. Both the *gemtc* and *pcnetmeta* packages require the use of a Bayesian software program (e.g., OpenBUGS or JAGS) to generate MCMC samples, though users are not required to work with that software directly, beyond installation. One shortcoming of all three R packages is that none of them currently perform network meta-regression to adjust for the effects of trial-level covariates, while they can be performed using other software programs [33], [34].

Table 3 presents the tools available to check assumptions and assess goodness-of-fit. In the case of NMA, statistical and visual tests are available to investigate violations of the assumptions of homogeneity and consistency. Methods to explore violations are available in *gemtc* and *netmeta* packages. Both the *gemtc* and *netmeta* packages include proposed global and pairwise indices for quantifying heterogeneity and inconsistency. To assess the goodness of fit of the model, the Deviance Information Criterion (DIC) is used in a Bayesian setting [35], and is available in both *gemtc* and *pcnetmeta* packages. The Akaike Information Criterion (AIC) is most widely used in a frequentist setting, but is not available in the *netmeta* package.

Table 4 shows the inferential and reporting capabilities of the three packages. Future investigators may benefit from knowing which specific summary measures are available from the different packages, given that some summary measures may be well-known or expected in certain fields. Relative effects are available from all packages, and rank probabilities, commonly reported in the Bayesian setting, are available in both *gemtc* and *pcnetmeta*. However, neither package has an in-built function for plotting the surface under the cumulative ranking probabilities (SUCRA) [36]. Although some work has been done investigating how ranks can be calculated in a frequentist setting [10], the frequentist package netmeta currently does not have this functionality. Several tools for displaying results are available from the various packages. The application to diabetes data presented below provides some context to display the various outputs available from each package.

### Application using Diabetes Data

**Figure 1:**
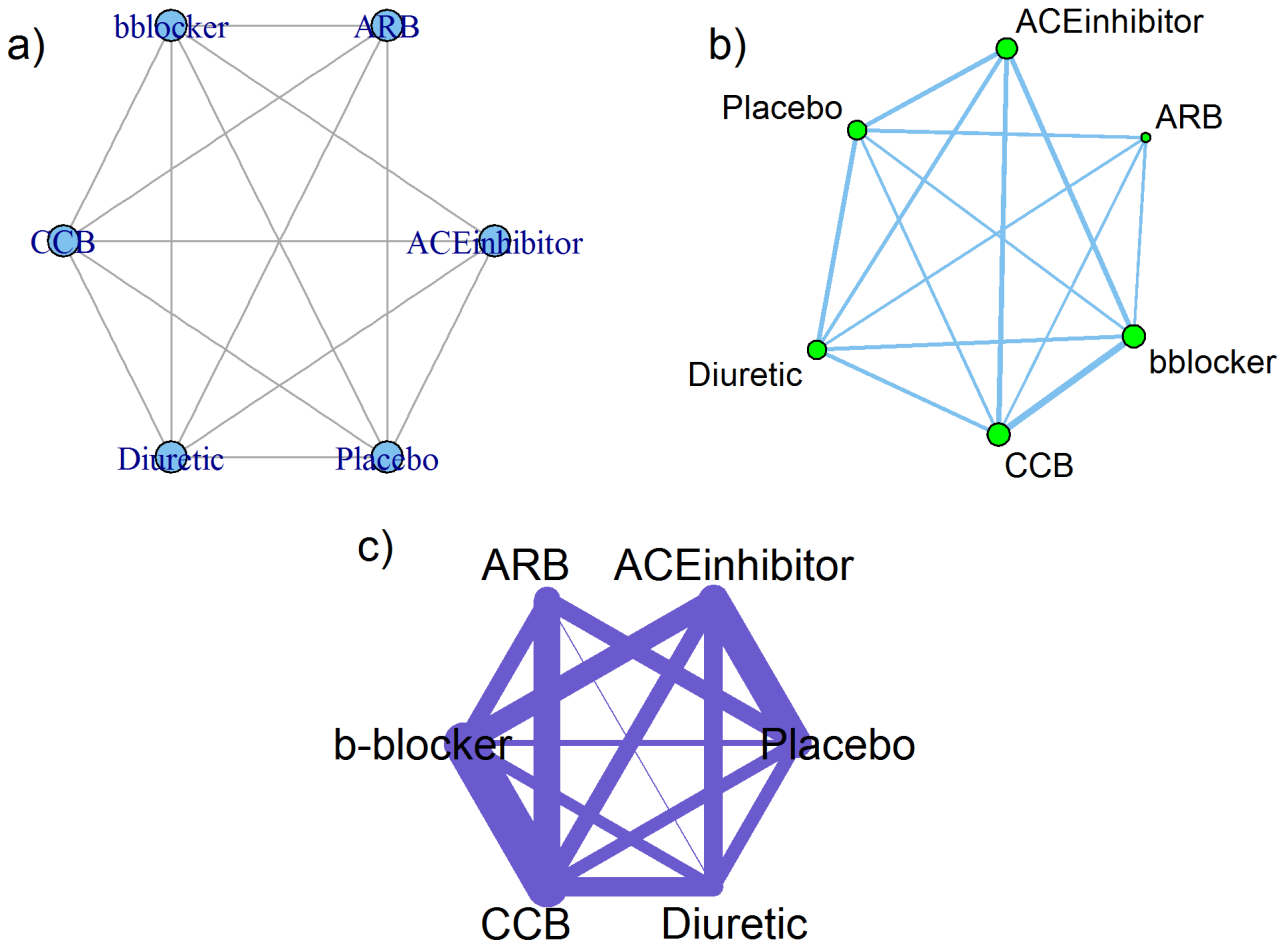
Network plots created by R packages a) *gemtc*, b) *pcnetmeta*, and c) *netmeta*.

We used all three packages to apply NMA to a previously published network dataset that compared the effects of six treatments for hypertension on the incidence of diabetes [27]. Treatment names and labels are summarized in Table 5. The network data set consists of arm-level summary data (i.e., number of events and total subjects randomized in each arm), with a total of 154,176 hypertensive patients in 22 trials (of which 18 were 2-arm and four were 3-arm trials). Of these, 10,962 patients developed diabetes during the follow up of time of the individual trials. To perform NMA, the arm-level summary data were used in *gemtc* and *pcnetmeta* packages. As *netmeta* package uses contrast-level data as input, we used the external function *escalc* available in the R package *metafor* [37] to convert the arm-level data into contrast-level data in each trial (relative treatment effect, namely, log-odds ratio) for the *netmeta* package. A plot of the network immediately after data input is available in the *gemtc* and *pcnetmeta* packages, and are displayed in Figs. 1a) and 1b), respectively.

**Table 5:** List of Treatment Reference Numbers for Diabetes Data.

Treatment Number	Treatment Name
1	ACE Inhibitor (ACE)
2	ARB
3	Beta-blocker (bblocker)
4	CCB
5	Diuretic
6	Placebo

For model selection, whenever available or applicable we applied the same or similar settings to all three packages. For example, we used a random-effects (RE) model assumption with a common heterogeneity parameter. In the *gemtc* and *pcnetmeta* analyses, we used non-informative priors for model parameters, and ran MCMC sampling for four chains, where first 100,000 posterior samples (burn-in period) were discarded and then another 100,000 posterior samples were saved in an interval of 10 in each chain. Convergence was attained based on visual inspection of time-series plots and using the Brooks-Gelman-Rubin test [38]. Once the model was run, a plot of the network was available from the *netmeta* package as displayed in Fig. 1c.

**Figure 2:**
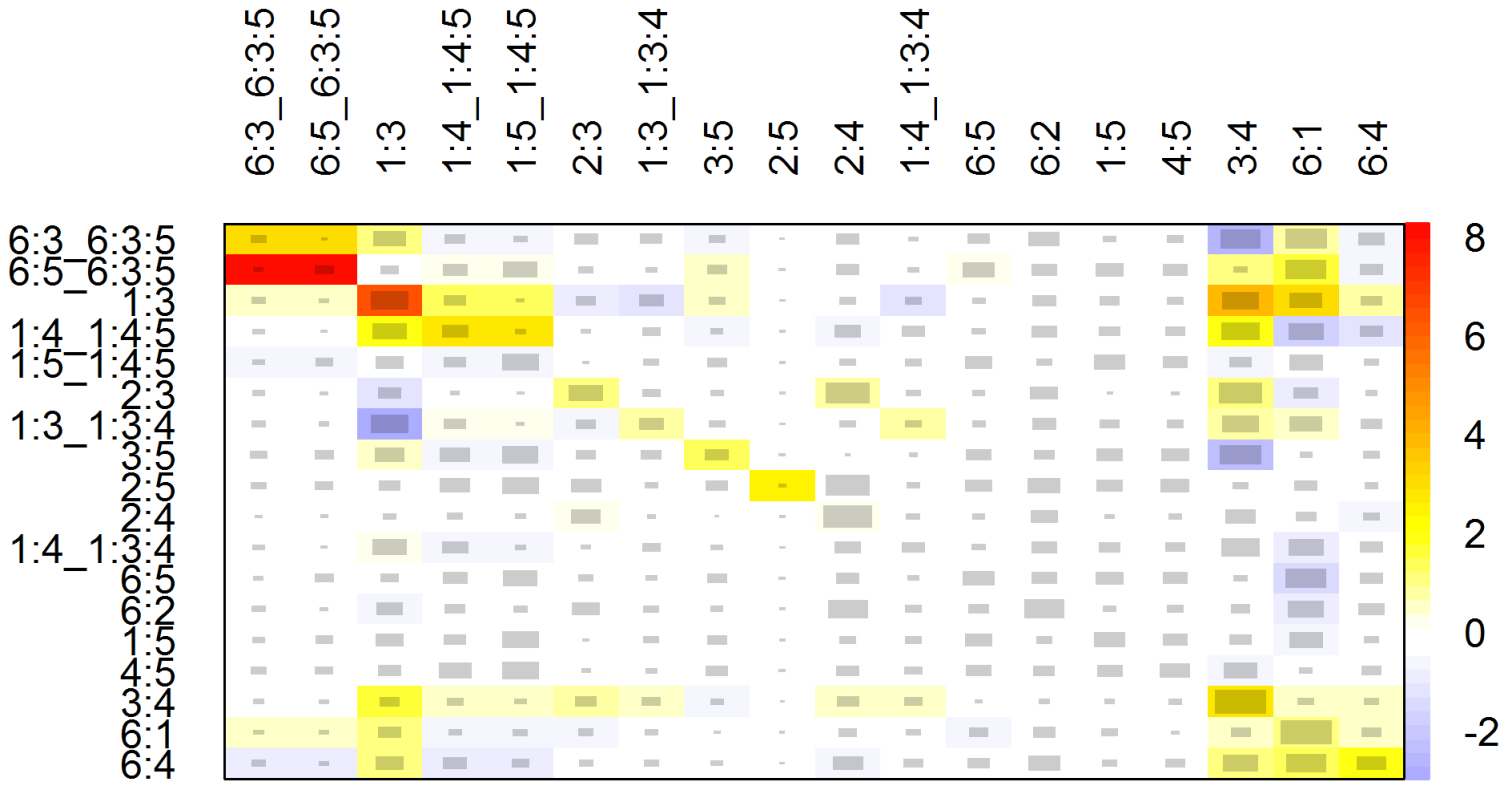
Inconsistency-detecting heat map function *netheat* from the *netmeta* package applied to the diabetes data set.

Using each package, we applied the available functions to test assumptions of homogeneity and consistency. The *pcnetmeta* package is not designed to assess consistency nor quantify heterogeneity in its output, whereas it does allow incorporating heterogeneity in the network assuming common or different heterogeneity levels. From *gemtc*, the global heterogeneity parameter, I^2^, was 43.98% with a range of 0 to 71.7% for the pairwise heterogeneity measures while the pairwise p-values for inconsistency, obtained via back-calculation, ranged from 0.02 to 0.99. The *netmeta* package provides a single heterogeneity/inconsistency I^2^ value of 57.61% from a Q statistic for the overall network of 49.54 which has a chi-square distribution with 21 degrees of freedom and yields a p-value of 0.0004. The Q statistic is further decomposed into heterogeneity and inconsistency components, valued at 28.3 and 21.2, respectively. Pairwise p-values for heterogeneity ranged from 0.03 to 1. Further, to identify inconsistency visually, the *netmeta* package provides a heat mapping function that is displayed in Fig. 2.

To assess model fit, both *gemtc* and *pcnetmeta* provide values for the DIC, which are 411.92 and 427.10, respectively. However, it is not appropriate to compare their model fits directly as the different modeling approaches make such comparison invalid.

The estimated odds ratios yielded from each of the three packages are included in Table 6 using the placebo as the reference treatment. For the Bayesian packages, median posterior values have been reported here. All three packages produce similar estimates and confidence intervals, with any differences probably attributable to the modeling approaches.

**Table 6:** Estimates of odds ratios and 95% credible or confidence intervals of treatment effects in Diabetes data by three R packages.

Effects	*gemtc*	*Pcnetmeta*	*netmeta*
	OR (95% CrI)	OR (95% CrI)	OR (95% CI)
Trt 1 vs. 6	0.89 (0.76, 1.04)	0.89 (0.82, 0.97)	0.88 (0.77, 1.02)
Trt 2 vs. 6	0.82 (0.68, 1.00)	0.81 (0.73, 0.90)	0.83 (0.69, 0.99)
Trt 3 vs. 6	1.25 (1.05, 1.50)	1.21 (1.10, 1.33)	1.24 (1.05, 1.46)
Trt 4 vs. 6	1.05 (0.89, 1.26)	1.00 (0.92, 1.10)	1.05 (0.89, 1.22)
Trt 5 vs. 6	1.34 (1.13, 1.63)	1.25 (1.13, 1.38)	1.33 (1.12, 1.57)

**Figure 3:**
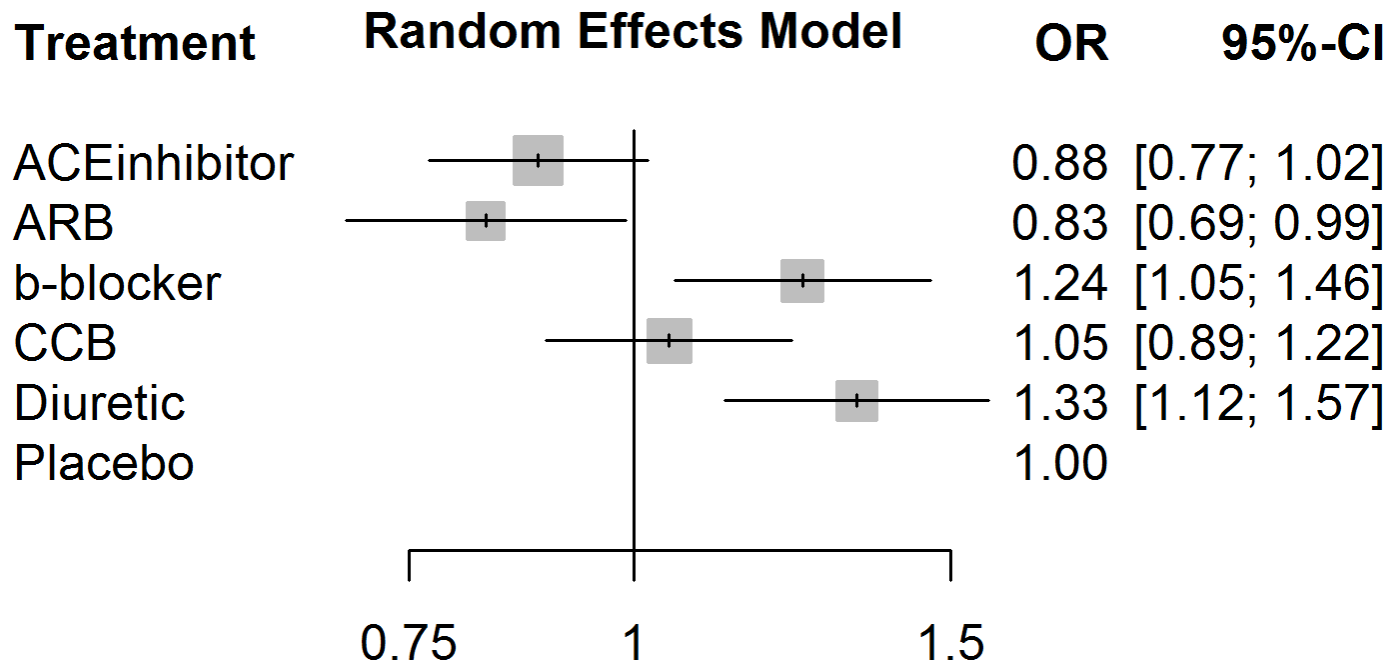
A forest plot of the estimates of odds ratios between each treatment and the reference placebo created using the *gemtc* R package and diabetes data.

**Figure 4:**
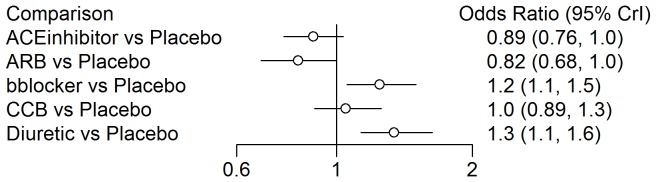
A sample of the detailed comparison-wise forest plots available from the *gemtc* R package outlining odds ratio estimates from contributing studies, direct evidence and indirect evidence using treatments 5 (diuretic) and 6 (placebo) from the diabetes data.

**Figure 5:**
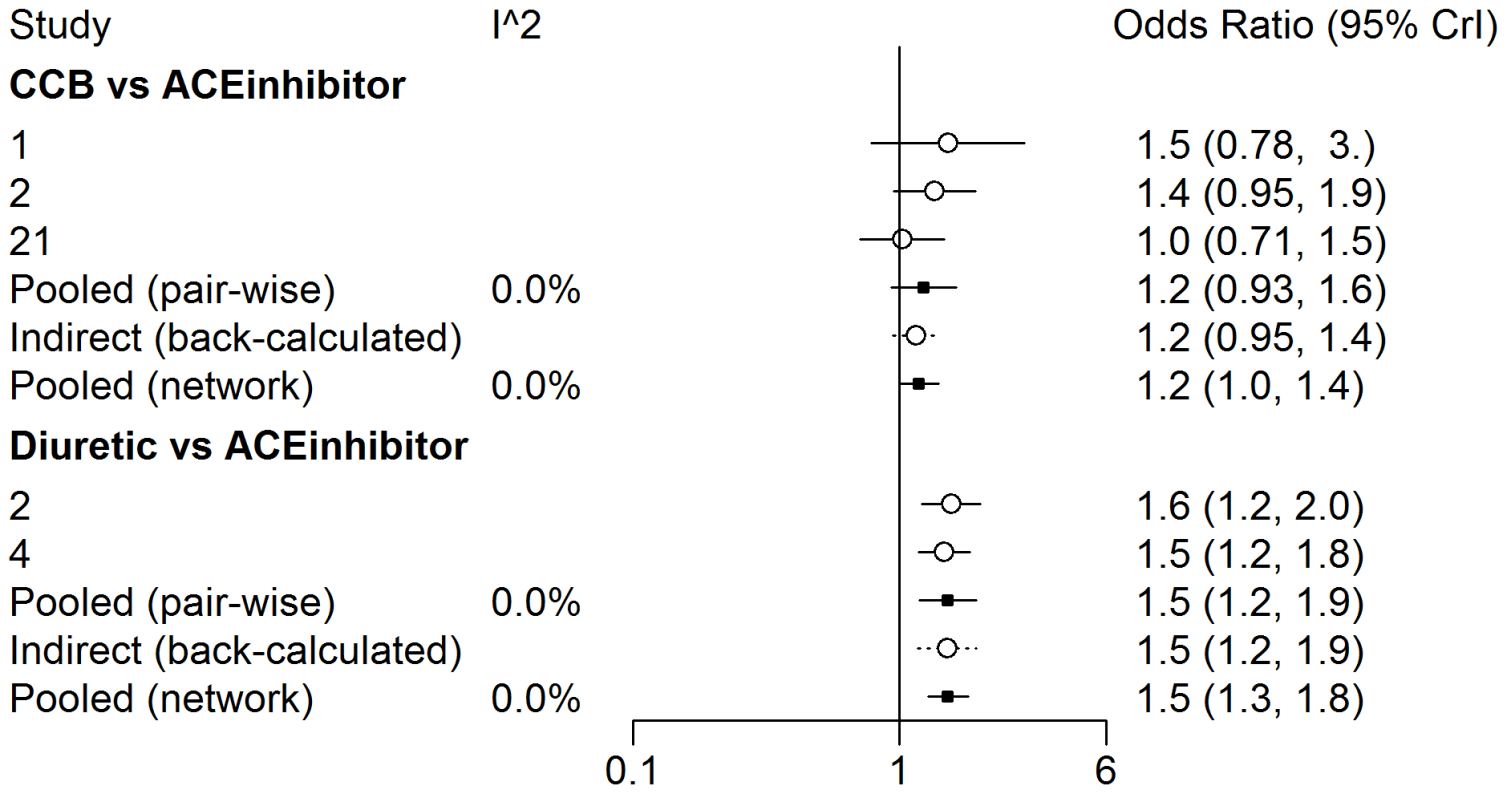
A forest plot of the estimates of odds ratios between each treatment and the reference placebo created using the *netmeta* R package and diabetes data.

**Figure 6:**
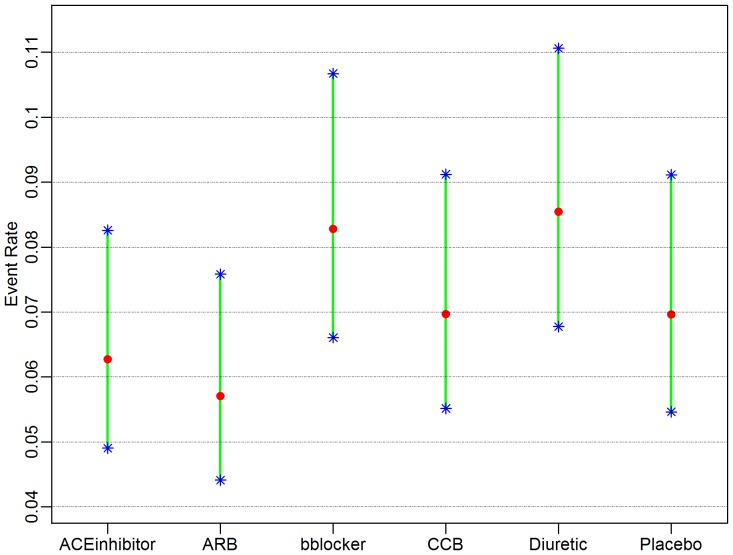
A confidence interval plot from the *pcnetmeta* R package displaying estimates of the event rates for all treatments in the diabetes dataset.

**Figure 7:**
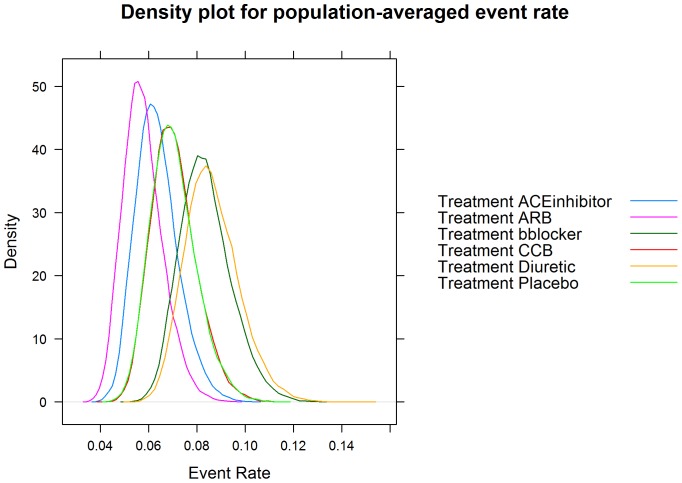
A density plot from the *pcnetmeta* R package displaying posterior densities for estimates of the event rates for all treatments in the diabetes dataset.

In order to introduce the reader to the various outputs available from each of the three R packages, several figures are included. A forest plot available from the *gemtc* package provides the pairwise estimates of odds ratios, shown in Fig. 3, and also includes a visual breakdown of each pairwise comparison; where treatments 5 (‘Diuretic’) vs. 6 (‘Placebo’) are illustrated in Fig. 4. From *netmeta*, Fig. 5 displays a forest plot of pairwise odds ratio estimates using the placebo as reference treatment. From *pcnetmeta*, a confidence interval plot of estimated event rates of the six treatments is illustrated in Fig. 6. Density plots of the event rate parameters are also created, seen in Fig. 7.

**Figure 8:**
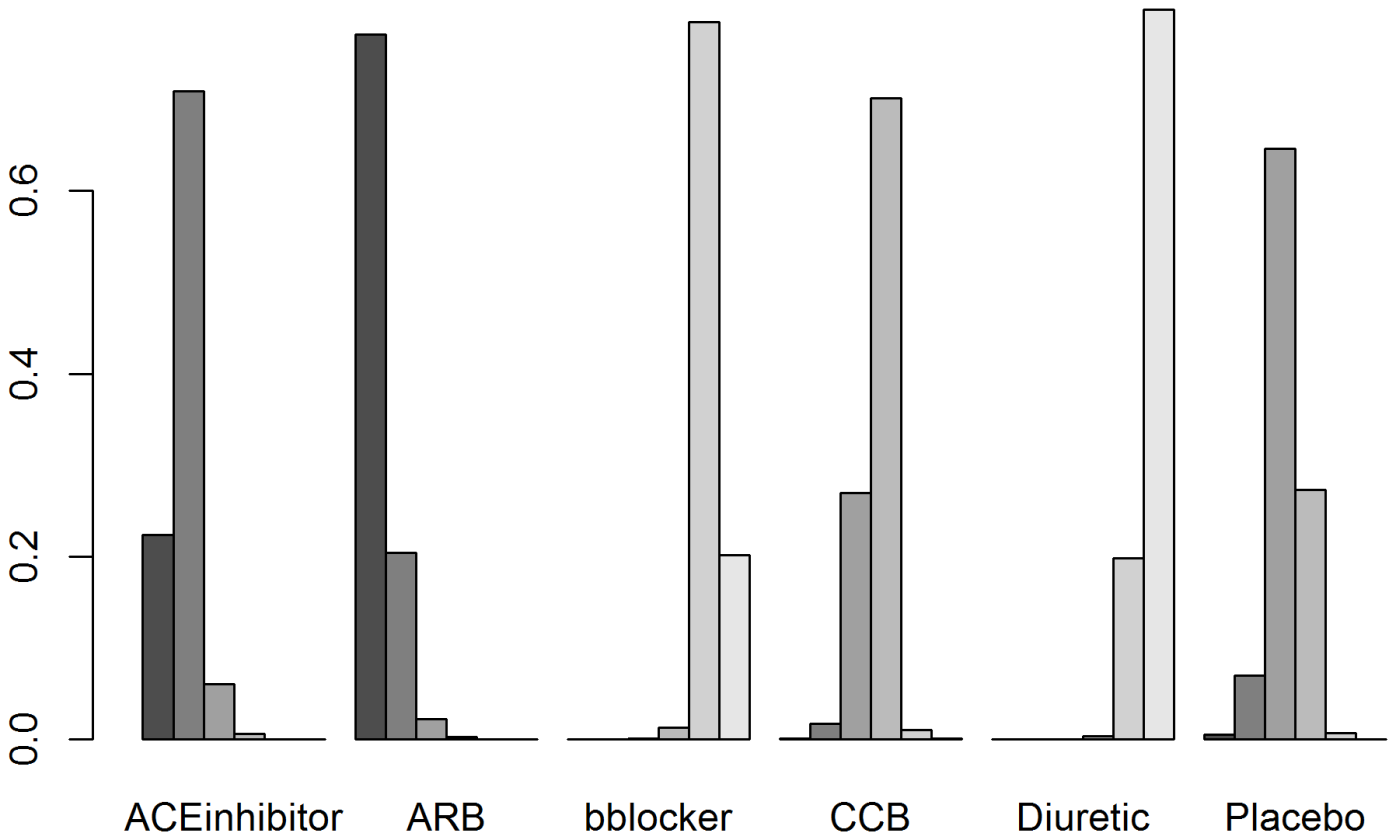
A rank plot created using the *rankogram* function from the *gemtc* R package applied to the diabetes dataset illustrating empirical probabilities that each treatment is ranked 1^st^ through 6^th^ (left to right).

For rankings, the *gemtc* package provides a matrix of the treatment rank probabilities displayed in Table 7, as well as a plot of the rank probabilities displayed in Fig. 8. The 1^st^ rank probabilities are estimated by *pcnetmeta* and available in Table 8. The two sets of rank probabilities yield the same ordering of the treatments.

**Table 7:** Rank probability matrix displaying estimated ranks of treatments from the Diabetes dataset obtained from the *gemtc* package.

Treatment Number	Treatment Name	Best	2^nd^	3^rd^	4^th^	5^th^	6^th^
1	ACE Inhibitor	0.2199	0.7132	0.0618	0.0051	0.0000	0.0000
2	ARB	0.7738	0.2025	0.0208	0.0028	0.0001	0.0000
3	Beta-blocker	0.0000	0.0000	0.0007	0.0145	0.7871	0.1978
4	CCB	0.0007	0.0182	0.2715	0.6984	0.0109	0.0004
5	Diuretic	0.0000	0.0000	0.0002	0.0032	0.1950	0.8016
6	Placebo	0.0056	0.0662	0.6451	0.2760	0.0069	0.0002

**Table 8:** Estimated 1^st^ rank probabilities of treatments from the Diabetes dataset obtained from the *pcnetmeta* package.

Treatment Number	Treatment Name	Probability Best Treatment
1	ACE Inhibitor	0.038
2	ARB	0.962
3	Beta-blocker	0.000
4	CCB	0.000
5	Diuretic	0.000
6	Placebo	0.000

## Conclusions

The three R packages presented in this paper offer different and often complementary features to perform all aspects of NMA. One or more of these packages could be used to plot the network, generate a model, detect heterogeneity or inconsistency in the network and incorporate them into the estimation, and finally plot the estimated effects sizes or ranks probabilities.

*Gemtc* and *netmeta* are comprehensive packages that employ Bayesian and frequentist techniques, respectively, to carry out NMA with flexibility, diligence and expertise. We have tried to summarize the key features important to any researcher conducting a NMA, leaving some of the functionality that extends beyond what is listed in the tables to the reader to investigate (e.g. *netmeasures* and *decomp.design* functions from *netmeta; mtc.anohe* and *mtc.nodesplit* functions from *gemtc*). To make full use of these packages, researchers are encouraged to read about the models employed and understand all of the modeling options provided by these two packages. In contrast, *pcnetmeta* is not designed to be fully comprehensive, but instead to provide a small set of functions that make the modeling process very simple for the user by leaving out many options. This Bayesian software will allow researchers with binary arm-level data to yield key outputs including a network plot and pooled events rates or relative effect estimates.

With two exceptions – meta-regression and SUCRA calculations – all of the functionalities we found important to the NMA modeling process were available in one or more of the three R packages investigated. We mention these shortcomings to encourage package developers to consider work in these areas. With respect to computational resource usage, it was difficult to make a fair comparison across the three R packages because they differ significantly in their default behavior, i.e., some of them run multiple tasks by default while others allow specification of targeted tasks (e.g., generating a diagnostic plot separately). Our general observation is that the “netmeta” package is the fastest and the other two have comparable computational resource usage. The NMA packages are constantly evolving with new features added in every update. What we have presented here will be updated over time, but we hope this will guide new researchers trying to employ NMA techniques to understand both the process and the tools that should be employed regardless of the package selected. The R codes that were used to generate the results reported in this paper are available at http://beyene-sigma-lab.com/code.html.
