# Correction: Network Meta-Analysis Using R: A Review of Currently Available Automated Packages

**DOI:** 10.1371/journal.pone.0123364

**Published:** 2015-04-02

**Authors:** The PLOS ONE Staff

The order of Figs. 3, 4, and 5 are incorrectly switched. The image that appears as Fig. 3 should be Fig. 5, the image that appears as Fig. 4 should be Fig. 3, and the image that appears as Fig. 5 should be Fig. 4. The Figure legends appear in the correct order. Additionally, the caption for Fig. 4 is incorrect, and the description of Fig. 4 in the second sentence of the sixth paragraph under the subheading Application using Diabetes Data in the Results section is incorrect. The correct sentence is “A forest plot available from the *gemtc* package provides the pairwise estimates of odds ratios, shown in Fig. 3, and also includes a visual breakdown of each pairwise comparison where two of the pairwise comparisons are illustrated in Fig. 4.” Please view Figs 3, 4, and 5, along with their correct legends here.

**Fig 3:**
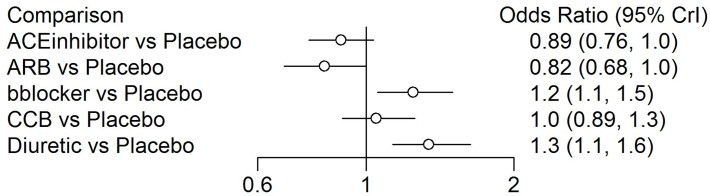
A forest plot of the estimates of odds ratios between each treatment and the reference placebo created using the *gemtc* R package and diabetes data.

**Fig 4:**
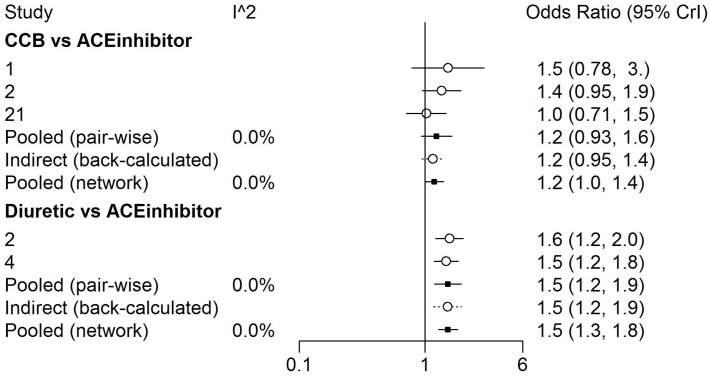
A sample of the detailed comparison-wise forest plots available from the *gemtc* R package outlining odds ratio estimates from contributing studies, direct evidence and indirect evidence using two of the pairwise comparisons from the diabetes data.

**Fig 5:**
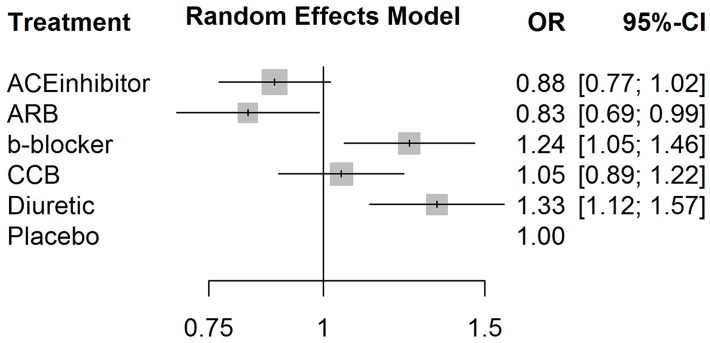
A forest plot of the estimates of odds ratios between each treatment and the reference placebo created using the *netmeta* R package and diabetes data.
